# Effect of B/N Doping on Enhanced Hydrogen Storage in Transition Metal-Modified Graphene: A First-Principles DFT Study

**DOI:** 10.3390/ma18194635

**Published:** 2025-10-08

**Authors:** Qian Nie, Lei Wang, Ye Chen, Zhengwei Nie

**Affiliations:** School of Mechanical and Power Engineering, Nanjing Tech University, Nanjing 211816, China; nieqian@njtech.edu.cn (Q.N.); gulin626@163.com (L.W.); chenye@njtech.edu.cn (Y.C.)

**Keywords:** B/N-doped, graphene, transition metal, hydrogen storage

## Abstract

Hydrogen energy is viewed as a promising green energy source because of its high energy density, abundant availability, and clean combustion results. Hydrogen storage is the critical link in a hydrogen economy. Using first-principles density functional theory calculations, this work explored the role of B and N in modulating the binding properties of transition metal-modified graphene. The hydrogen storage performance of Sc-, Ti-, and V-modified B-doped graphene was evaluated. Boron doping induces an electron-deficient state, enhancing interactions between transition metals and graphene. Sc, Ti, and V preferentially adsorbed at the carbon ring’s hollow site in B-doped graphene, with their binding energies being 1.87, 1.74, and 1.69 eV higher than those in pure graphene, respectively. These systems can stably adsorb up to 5, 4, and 4 H_2_ molecules, with average adsorption energies of −0.528, −0.645, and −0.620 eV/H_2_, respectively. The hydrogen adsorption mechanism was dominated by orbital interactions and polarization effects. Among the systems studied, Sc-modified B-doped graphene exhibited superior hydrogen storage characteristics, making it a promising candidate for reversible applications.

## 1. Introduction

As a potential energy carrier, hydrogen energy is characterized by high energy density, abundant reserves, and environmental friendliness [1,2]. Promoting the development of hydrogen energy is a key strategy for reducing fossil fuel dependency and achieving renewable energy goals [3]. Hydrogen energy utilization includes hydrogen production, storage, and application. Hydrogen production and application in hydrogen energy have been relatively mature. However, hydrogen energy storage and transportation face significant challenges. Whether hydrogen energy can be stored safely, efficiently, and economically is the key to hydrogen energy playing an important role in transforming the energy system [4,5]. Hydrogen storage technologies include high-pressure gas storage, low-temperature liquefaction, and solid-material storage. Among solid materials, carbon-based nanomaterials have received substantial attention due to their safety and efficiency [6,7].

Previous studies have focused on developing low-cost and lightweight carbon-based materials, such as graphite [8], activated carbon [9], graphene [10], and carbon nanotubes [11,12]. These materials exhibited high specific surface areas and large pore volumes, providing additional hydrogen adsorption sites. Studies have shown that the interactions between pure graphene and hydrogen molecules were weak, relying on physical adsorption. Graphene could reversibly adsorb and desorb hydrogen molecules under ambient temperature and pressure conditions [13], offering substantial hydrogen storage potential. However, its adsorption efficiency was low (<1.0 wt.%) [14,15], making it unsuitable for hydrogen storage at room temperature. To improve hydrogen storage capacity, metal catalysts such as Pt, Pd, and Ni were typically loaded onto carbon-based materials through chemical or physical doping [16,17]. Seenithurai et al. [18] calculated the hydrogen storage properties of Li-modified graphene using the Dmol^3^ module. The results showed that the binding energy of Li-decorated double-carbon vacancy graphene (DVG) was 4.04 eV, much higher than that of Li-decorated pure graphene. When Li was modified on both sides of DVG, the weight storage capacity reached 7.26 wt.%, and the binding energy was 0.26 eV/H_2_. Ataca et al. [19] found that Ca-modified 4 × 4 graphene can adsorb up to 5 H_2_ molecules. Similarly, Wang et al. [20] reported that Ca-modified graphene nanotubes (GNT) had a hydrogen storage capacity of 7.44 to 8.96 wt.%, making them a viable option for hydrogen storage. However, the metal was easy to agglomerate because of its high cohesion energy, resulting in reduced hydrogen storage capacity [21]. As this challenge occurred frequently in practical applications and experiments, it was imperative to seek effective solutions. The ability of light metal complexes (transition metals) to absorb hydrogen was demonstrated in relevant studies [22,23]. Heteroatom doping can enhance the binding energy between the transition metal and the graphene substrate, thereby improving the hydrogen storage performance [24,25].

Transition metals (e.g., Sc, Ti, and V) have been well demonstrated to enhance the adsorption of H_2_ by carbon-based materials [26,27,28]. Huo et al. [29] found that B doping in graphene can significantly increase the metal–substrate interaction and prevent Ti-metal clusters’ formation. Wang et al. [30] used density functional theory (DFT) to study the optimal geometry and hydrogen storage of Sc-modified graphene. Their study identified that the configuration with the highest hydrogen storage capacity featured double Sc atoms positioned on opposite sides of the center of the boron–carbon hexagon. Theoretically, this configuration demonstrated a hydrogen storage capacity of 9.13 wt.%. However, if hydrogen storage is achieved by adsorbing Sc atoms on both sides of the PG, it may lead to instability of the structure.

Chen et al. [31] studied hydrogen storage on Pt_4_ clusters supported on pure, B-doped, and N-doped graphene sheets through DFT calculations. The results showed that B or N doping in graphene significantly enhanced the interactions between the substrate and Pt_4_ clusters. When metal nanoparticles were adsorbed on heteroatom-doped graphene, the adsorption energy of metal atoms was significantly increased, thereby preventing their aggregation [32]. Wu et al. [33] investigated B-doped graphene’s hydrogen storage performance using DFT. The results show that carbon atoms adjacent to the B atom significantly enhanced hydrogen adsorption capability, allowing catalytic metals to bind more strongly. Lueking et al. [34] investigated the effect of heteroatoms on binding energy and mobility on graphene. Boron-doped graphene and hydroxyl fossil ink may meet the thermodynamic and kinetic constraints of a reversible room-temperature hydrogenation reaction. It has been reported that the N element could improve the microstructure of the substrate and provide more hydrogen adsorption sites on the carbon-based material. Luo et al. [35] explored the N-doped activated carbon’s microporous structure. The research found that N doping can increase the activated carbon’s surface area to 3485 m^2^/g. Zhao et al. [36] doped N onto Pt-modified activated carbon’s surface using a hydrothermal method. The results showed that N doping enhanced Pt nanoparticles’ pore size and dispersion. Researchers have found that B and N doping played a crucial role in enhancing hydrogen storage capacity [37,38].

In this study, B- and N-modified doped graphene (BGr and NGr) models were designed using first-principles DFT calculations. The interactions and hydrogen storage performance of transition metal-modified B-doped graphene, as well as the effects of doping on enhanced hydrogen storage, were systematically studied. The optimal transition metal-modified position and binding energy were obtained. The hydrogen storage mechanism of transition metal-modified doped graphene was revealed.

## 2. Simulation Methods

All calculations in this work were conducted using first-principles DFT calculations, specifically with the CASTEP module [39]. The exchange-correlation energy was calculated within the Perdew–Burke–Ernzerhof generalized gradient approximation (GGA-PBE). The ultrasoft (US) pseudopotential expressed in reciprocal space was utilized to enhance computation efficiency and accuracy. A cutoff energy of 500 eV was set for the overall plane-wave basis set expansion. The Brillouin zone was sampled using a Monkhorst–Pack grid; and the k-point was assigned to 3 × 3 × 1 for our calculations. All calculations included spin polarization. Given that the GGA function may underestimate weak adsorption energy (Van der Waals), Grimme dispersion correction in DFT-D was utilized to improve the calculations [40,41]. The convergence tolerances for the optimized geometric structure were established as follows: energy at 2.0 × 10^−5^ eV/atom, maximum force at 0.02 eV/Å, maximum stress at 0.04 GPa, and maximum displacement at 0.002 Å. We selected an electronic self-consistent field (SCF) tolerance of 1.0 × 10^−6^ eV/atom. The graphene supercell size was chosen as 5 × 5 × 1, with a 20 Å vacuum layer included to reduce interactions between adjacent layers.

The binding energy ($\Delta E_{b}$) of transition metal atom (TM) modification on pure graphene (Gr) is calculated using the following equation:

(1) $$\Delta E_{b}\;=\;[E_{\mathrm{TM}+\mathrm{Gr}}-E_{\mathrm{Gr}}-E_{\mathrm{TM}}]$$

where $E_{\mathrm{TM}+\mathrm{Gr}}$, $E_{\mathrm{Gr}}$, and $E_{\mathrm{TM}}$ represent the total energy of a transition metal atom adsorbed on a Gr supercell, the total energy of the Gr supercell, and the total energy of a free TM, respectively.

The adsorption energy ($\Delta E_{\mathrm{ad}}$) and average adsorption energy ($\Delta {\bar{E}}_{\mathrm{ad}}$) of H_2_ molecules on transition metal-modified B-doped graphene (TM-BGr) are calculated using the following equations, respectively:

(2) $$\Delta E_{\mathrm{ad}}\;=\;[E_{{nH}_{2}+\mathrm{TM}+\mathrm{BGr}}-E_{{(n-1)H}_{2}+\mathrm{TM}+\mathrm{BGr}}-E_{H_{2}}]$$

(3) $$\Delta {\bar{E}}_{\mathrm{ad}}=[E_{{nH}_{2}+\mathrm{TM}+\mathrm{BGr}}-E_{\mathrm{TM}+\mathrm{BGr}}-{nE}_{H_{2}}]/n$$

where $E_{{nH}_{2}+\mathrm{TM}+\mathrm{BGr}}$, $E_{{(n-1)H}_{2}+\mathrm{TM}+\mathrm{BGr}}$, $E_{\mathrm{TM}+\mathrm{BGr}}$, and $E_{H_{2}}$ represent the total energy of nH_2_ molecules adsorbed on TM-BGr, the total energy of (*n*−1)H_2_ molecules adsorbed, the total energy of TM-BGr, and the total energy of one free H_2_ molecule, respectively.

The gravimetric hydrogen density, also known as the hydrogen storage capacity (HSC), can be calculated using the following equation [42]:

(4) $$H_{2}\;(\mathrm{wt}.\%)=\frac{\;n\;\times {\;M}_{H_{2}}}{{n\;\times \;M}_{H_{2}}\;+{\;M}_{\mathrm{host}}}\;\times \;100$$

where the $M_{H_{2}}$ and $M_{\mathrm{host}}$ represent hydrogen’s and host material’s molar mass, respectively, and the *n* refers to the number of stored H_2_ molecules.

## 3. Results and Discussion

### 3.1. Sc, Ti, and V Atoms Modify the Structure of Gr, BGr, and NGr

**Figure 1:**
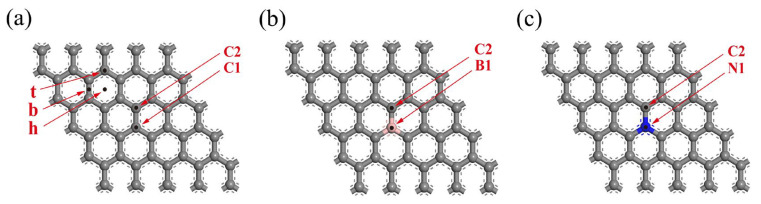
Geometrically optimized structures of (**a**) pure graphene, (**b**) B-doped graphene, and (**c**) N-doped graphene (h, b, and t refer to hollow sites, bridge sites, and top sites, respectively; pink for the B atom and blue for the N atom).

**Figure 2:**
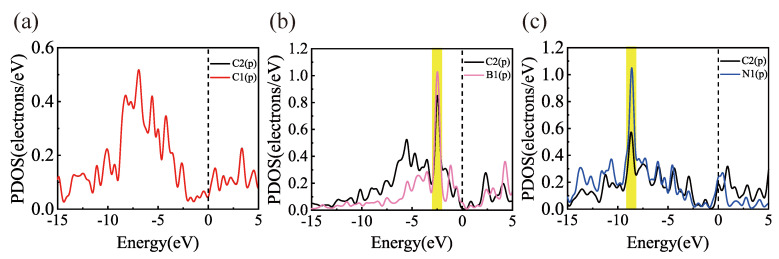
PDOS diagrams for (**a**) pure graphene, (**b**) B-doped graphene, and (**c**) N-doped graphene.

To compare and analyze the results in the literature and verify the correctness of the calculation model, the geometrically optimized structures of Gr, BGr, and NGr are first calculated in this paper, as shown in Figure 1. The C1–C2 bond length in pristine graphene is 1.43 Å. Upon B and N doping, the calculated B1–C2 and N1–C2 distances increase to 1.49 Å and 1.44 Å, respectively, in good agreement with literature results [27,43,44,45,46]. To explain the interactions of doped B and N in graphene, the partial density of states (PDOS) is calculated, as shown in Figure 2. For Gr, the p orbitals of C1 and C2 have a strong hybridization. After B and N doping, the hybrid range of the p orbitals of B1 and C2 is between −2.0 eV and −3.0 eV. The hybrid range of the p orbitals of N1 and C2 is between −8.0 eV and −9.0 eV. As reported previously [47], this agrees with previous findings. Based on the above results, the calculation model, the method, and the parameter settings used in this study are reliable.

**Figure 3:**
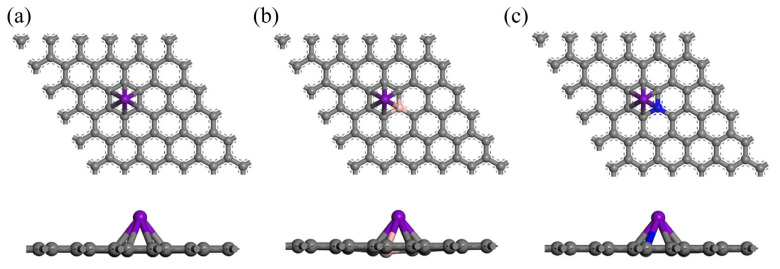
Geometrically optimized structures of transition metal-modified (**a**) pure graphene, (**b**) B-doped graphene, and (**c**) N-doped graphene (purple indicates transition metal).

Transition metal atoms have three possible binding sites on graphene: the bridge site (B), the hollow site (H), and the top site (T), as shown in Figure 1. Further, the geometric structure optimization of Sc, Ti, and V models in pure, B-doped, and N-doped graphene is carried out, as shown in Figure 3. Sc, Ti, and V modifications have the highest binding energies at the pure, B-doped, and N-doped graphene hollow sites, which are in substantial agreement with the reported behavior of transition metal atoms (Sc, Ti, and V) at graphene hollow sites [48,49,50]. According to calculations and analysis by Equation (1), the binding energies of Sc, Ti, and V at the hollow site of pure graphene are −1.34, −1.80, and −1.30 eV, respectively. These energies are very close to the results reported by Lebon et al. [50]. The vertical distances of Sc, Ti, and V from the substrate at the hollow site of pure graphene are 1.893, 1.842, and 1.820 Å, respectively, closely matching the results reported by Manade et al. [48].

The binding energies of different transition metals with graphene, doped graphene, and other relevant parameters are listed in Table 1. After introducing B element on the surface of pure graphene, the interactions between Sc, Ti, and V transition metal elements and the base material are increased by 1.87, 1.74, and 1.69 eV, respectively, compared with pure graphene. This result demonstrates that the introduction of the B element effectively increases the binding energy between the transition metal and graphene, making the metal atoms more stable. Compared with pure graphene, doping with B and N elements causes changes in the TM-C bond length. The Hirshfeld charges of Sc-, Ti-, and V-modified the B-doped graphene are significantly increased compared to pure graphene. This result indicates that B doping transfers more electrons from the transition metal to the graphene material, significantly enhancing its binding energy. On the other hand, the Hirshfeld charges of N-doped graphene are similar to those of pure graphene, and the incorporation of N has little effect on improving metal-graphene interaction. Therefore, B doping can effectively enhance the interaction between the metal and the graphene.

**Figure 4:**
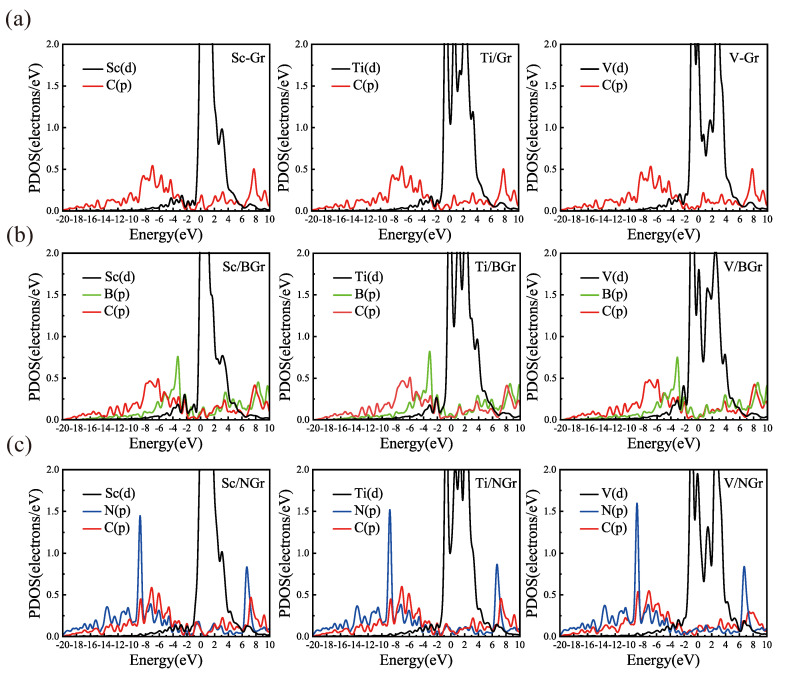
PDOS diagrams of Sc-, Ti-, and V-modified (**a**) pure graphene, (**b**) B-doped graphene, and (**c**) N-doped graphene systems.

As shown in Figure 4, PDOS of Sc-, Ti-, and V-modified pure, B-doped, and N-doped graphene systems are calculated. It can be seen from the figure that there is a specific orbital hybridization between transition metals and pure/doped graphene. This result indicates that B and N doping change the electronic structure of pure graphene. In the pure graphene system, the 2p orbitals of C atoms and the 3d orbitals of Sc, Ti, and V show significant overlap at around −2.0 eV. This resonance effectively enhances the interactions between the metal and the base material. For B-doped and N-doped graphene, the Fermi level of the BGr shifts toward the valence band due to electron deletion near the B atom that is transferred to the carbon atom. While the N atom gains electrons from the neighboring carbon atom on graphene, the Fermi level of the NGr moves toward the conduction band. In addition, B-doped graphene has a more substantial resonance peak between the 2p orbitals of C and the transition metals in the range of −2.0 to −3.0 eV. Furthermore, this resonance peak is higher than that in pure and N-doped graphene, indicating that Sc, Ti, and V form stronger bonds with the B-doped graphene substrate. This result thoroughly verifies that B doping is the main reason for enhancing the binding of transition metal atoms to graphene.

**Figure 5:**
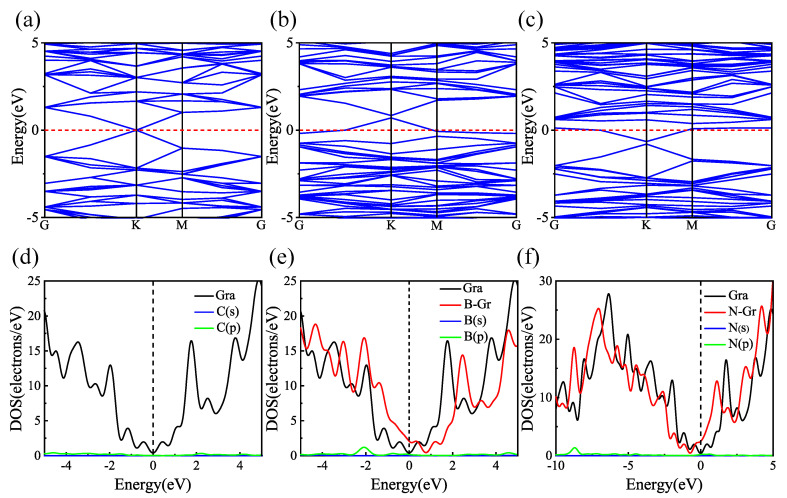
Band structure of (**a**) pure graphene, (**b**) B-doped graphene, and (**c**) N-doped graphene, and DOS diagrams of (**d**) pure graphene, (**e**) B-doped graphene, and (**f**) N-doped graphene.

To further study the effect of B and N doping on the properties of pure graphene, we calculate the band structure and DOS diagrams for pure graphene, as well as for graphene doped with a single B and N atom, as shown in Figure 5. It can be observed that, compared to pure graphene, the overall energy band shifts upward with B doping and downward with N doping. After doping, energy bands cross near the Fermi level. The electron-deficient property of B doping causes the system to exhibit p-type semiconductor behavior. In contrast, the electron-rich property of N doping causes the system to exhibit n-type semiconductor behavior. These results are consistent with the literature [51]. From the DOS diagrams, the peak of B-doped graphene shifts to the right in the range of −3.0~−2.0 eV due to the contribution of 2p orbitals of the B atom. In the range of −8.0~−9.0 eV, the peak of N-doped graphene shifts to the left due to the contribution of 2p orbitals of the N atom. The results show that doping with B and N atoms can improve pure graphene’s chemical activity. It should be pointed out that doped graphene is in an electron-deficient state due to the presence of the B atom, which can effectively increase the number of active sites on graphene [45,52]. Based on the above analysis, B-doped graphene is an active substrate for Sc, Ti, and V atom modification in the above systems. Accordingly, the following section only discusses H_2_ molecular adsorption in the transition metal-modified B-doped graphene system.

### 3.2. Hydrogen Adsorption of B-Doped Single Vacancy Defect Graphene Modified by Sc, Ti, and V Atoms

**Figure 6:**
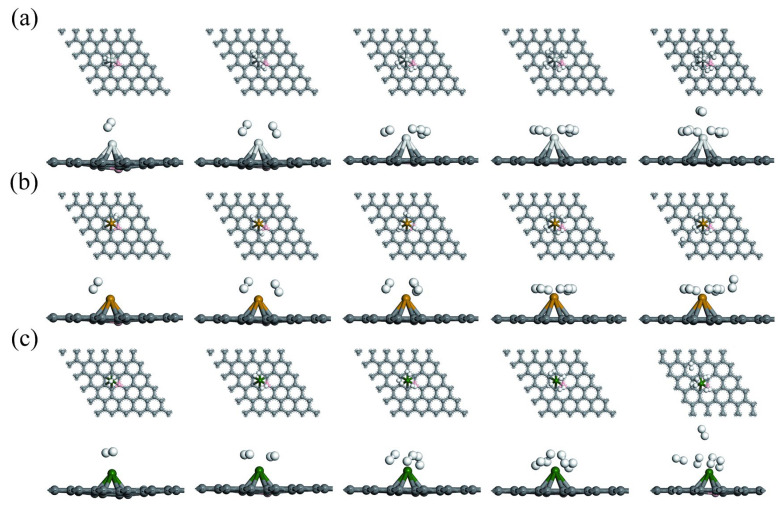
Geometrically optimized structure of adsorption H_2_ molecules by B-doped graphene modified with Sc, Ti, and V: (**a**) Sc-BGr, (**b**) Ti-BGr, and (**c**) V-BGr systems.

The geometrically optimized structures of the B-doped graphene system modified with Sc, Ti, and V after H_2_ adsorption are shown in Figure 6. Table 2 lists the adsorption energy and other parameters for three systems. It can be observed that these parameters change with the increasing number of H_2_ molecules. As shown in Figure 6, the first H_2_ molecule is preferentially adsorbed around Sc, Ti, and V, with binding energies of −0.75, −0.68, and −0.68 eV, respectively. Table 2 further reveals that the hydrogen molecules adsorb in a molecular form, with H-H bond lengths of 0.837 Å, 0.809 Å, and 0.823 Å, respectively. To study the maximum hydrogen storage capacity of three systems, H_2_ molecules are continuously added until the maximum adsorption capacity is reached. Due to the symmetry of H_2_ molecules’ bonding configuration, when the four H_2_ molecules are adsorbed, they are evenly distributed over Sc, Ti, and V, almost in the same plane. As shown in Figure 6a and Table 2, the fifth H_2_ molecule is adsorbed directly above the Sc atom, with an H-H bond length of 0.765 Å and a weak adsorption energy of −0.27 eV. From Figure 6b and Table 2, the fifth H_2_ molecule is adsorbed away from the central hole of the Ti atom, with a bond length of 0.752 Å and a weak adsorption energy of −0.0998 eV. This adsorption energy is well below the lower limit of the desired ideal adsorption energy (−0.2 eV) [29], indicating that the fifth H_2_ molecule undergoes only physical adsorption. Such weak adsorption can be negligible under practical conditions. According to Figure 6c and Table 2, the fifth H_2_ molecule is adsorbed at the central hole away from the V atom. Additionally, one H_2_ molecule is adsorbed above the V atom, separated from the other H_2_ molecules. This molecule has a bond length of 0.755 Å and a positive adsorption energy of 0.14 eV. This result indicates that the system after adsorption of this H_2_ molecule is thermodynamically unstable. In the above three systems, the fifth H_2_ molecule cannot undergo stable adsorption. A space limitation may result from an increase in H_2_ adsorption quantity [53] and the mutual repulsion between adsorbed H_2_ molecules.

**Figure 7:**
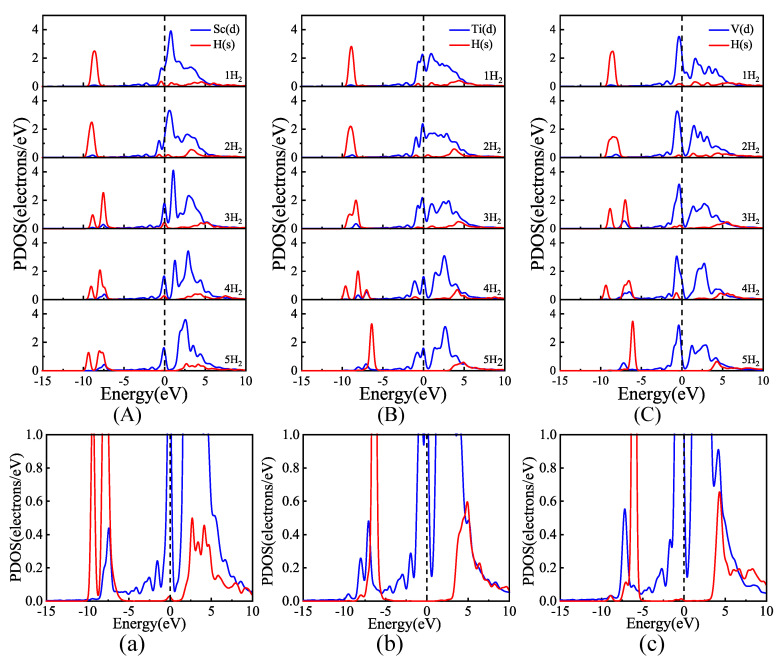
PDOS diagrams of H_2_ adsorption by B-doped graphene modified with Sc, Ti, and V: (**A**) Sc-BGr, (**B**) Ti-BGr, and (**C**) V-BGr systems; Partial enlarged PDOS diagrams of adsorption of the 5th H_2_ molecule by (**a**) Sc-BGr, (**b**) Ti-BGr, and (**c**) V-BGr systems.

To analyze the interactions between Sc, Ti, and V and the adsorbed H_2_ molecules in the studied system, the PDOS diagrams of H_2_ molecules and Sc, Ti, and V are provided in Figure 7. As seen in Figure 7, the 1s orbitals of H_2_ and the 3d orbitals of Sc, Ti, and V all exhibit orbital hybridization, suggesting a significant interaction between them. As shown in Figure 7A–C, after the adsorption of the third H_2_ molecule, the 1s orbitals of the H_2_ molecule exhibit broadening near −8.0, −8.0, and −7.5 eV, respectively. This broadening phenomenon indicates interactions between adsorbed H_2_ molecules. In addition, orbital coupling between the 1s orbitals of H_2_ and the 3d orbitals of Sc, Ti, and V can be observed in the range of −1.0~0.0 eV in the adsorption of the 1st to 4th H_2_ molecules. This result illustrates the strong hybridization of Kubas [54]. As shown in Figure 7a, the anti-bonding orbitals $\sigma^{*}$ of the fifth H_2_ molecule and the 3d orbital of the Sc atom exhibit weak orbital coupling at the Fermi level, suggesting that there is only a weak polarization interaction between them. As shown in Figure 7b,c, the anti-bonding orbitals $\sigma^{*}$ of the fifth H_2_ molecule and the 3d orbitals of the Ti and V atoms show the minimal band expansion or orbital coupling near the Fermi level, indicating no interactions between them.

**Figure 8:**
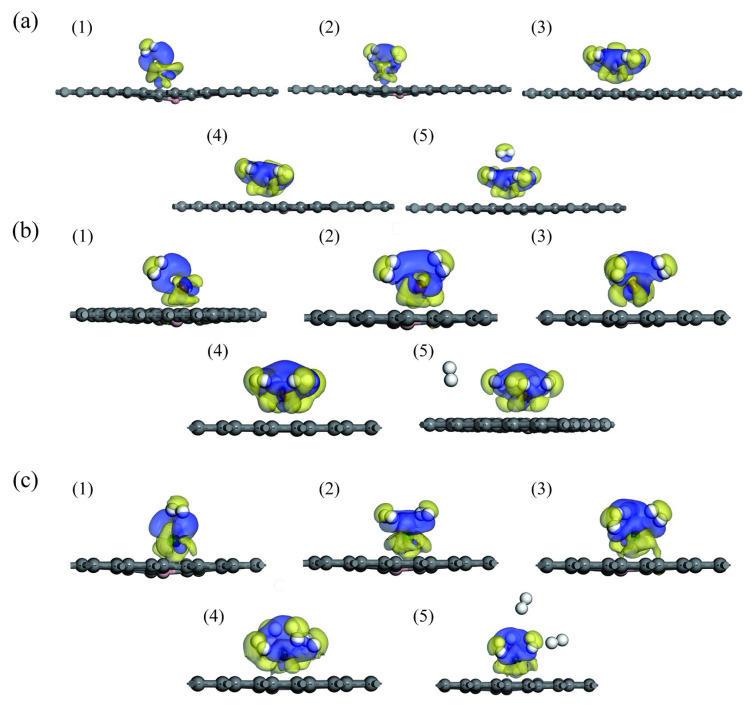
EDD diagrams of 1–5 H_2_ molecules adsorbed on B-doped graphene modified with Sc, Ti, and V: (**a**) Sc-BGr, (**b**) Ti-BGr, and (**c**) V-BGr systems (the light yellow and light blue isoplanes represent the regions where charge is lost and accumulated, respectively; and the isoplane unit is 0.02 e/Å^3^).

**Figure 9:**
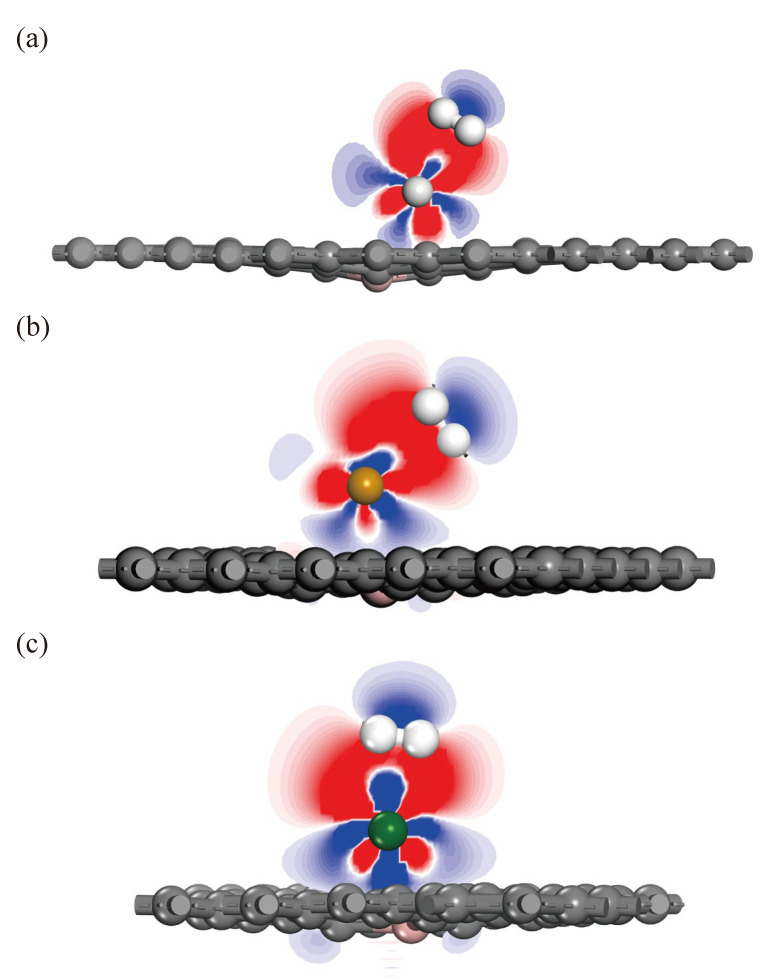
Two-dimensional planar EDD diagrams for adsorption of the first H_2_ molecule by B-doped graphene modified with Sc, Ti, and V: (**a**) Sc-BGr, (**b**) Ti-BGr, and (**c**) V-BGr systems (blue and red represent areas of charge loss and accumulation, respectively, with isosurface units of 0.02 e/Å^3^).

To better understand the adsorption mechanism, this paper analyzes the electronic difference density (EDD) diagrams for H_2_ adsorption in Sc-, Ti-, and V-modified B-doped graphene systems, as shown in Figure 8. The yellow and blue isoplanes visually show the regions of charge loss and accumulation, respectively, with isoplanes in units of 0.02 e/Å^3^. Mulliken charge layouts before and after each system’s adsorption of one H_2_ molecule are listed in Table 3. H_2_ molecules obtained more electron transfer in the Sc-BGr system, further confirming the results from the adsorption energy and PDOS analysis. Based on Mulliken layout analysis and Figure 8, transition metal atoms and the base material are positively and negatively charged, respectively, indicating that charge transfer exists between them. An electric field is generated between the transition metal atoms and the base material, which polarizes the H_2_ molecule. According to molecular orbital theory, in an isolated H_2_ molecule, the 1s orbitals of the two hydrogen atoms form σ bonding orbitals and $\sigma^{*}$ antibonding orbitals. By analyzing the EDD diagram, it is found that the charge reduction region lies between two H atoms, reflecting the σ orbital of the H_2_ molecule. The region around which the charge accumulates is the $\sigma^{*}$ orbital of the H_2_ molecule. Additionally, transition metal atoms have both accumulation and decrease regions around them. These results indicate that the σ orbital of the H_2_ molecule loses electrons, which are transferred to the transition metal atom with an unfilled d orbital. Simultaneously, electrons from the d orbital of the transition metal atom are transferred to the $\sigma^{*}$ antibonding orbital of the H_2_ molecule, forming a feedback bond. Figure 9 shows the two-dimensional planar EDD diagrams of the first H_2_ molecule adsorption in three systems, from which Kubas’ effect can be clearly observed. Therefore, the adsorption mechanism of H_2_ molecules in Sc-BGr, Ti-BGr, and V-BGr systems mainly involves two aspects. First, the orbital interactions between H_2_ and Sc, Ti, and V play a key role. Second, the electrostatic field generated between Sc, Ti, and V and the substrate material causes polarization of H_2_ molecules, which in turn creates a Coulomb attraction between the negatively charged H_2_ molecules and the positively charged Sc, Ti, and V.

As seen in Figure 8a(5), the charge gain and loss of the fifth H_2_ molecule are relatively small and independent, indicating that the adsorption strength of this H_2_ molecule is weak. Adsorption results from polarization caused by the electric field. As shown in Figure 8b(5), there is no charge accumulation or reduction around the fifth H_2_ molecule. Combined with the adsorption energy parameters of the H_2_ molecule in Table 2, this reveals that the adsorption strength of the fifth H_2_ molecule in the system is very weak. Similarly, as seen in Figure 8c(5), no charge accumulation or decrease is observed around the fifth H_2_ molecule. The adsorption energy data in Table 2 further suggest that the fifth H_2_ molecule cannot be adsorbed into the system.

### 3.3. Comparison of Hydrogen Storage Properties

**Figure 10:**
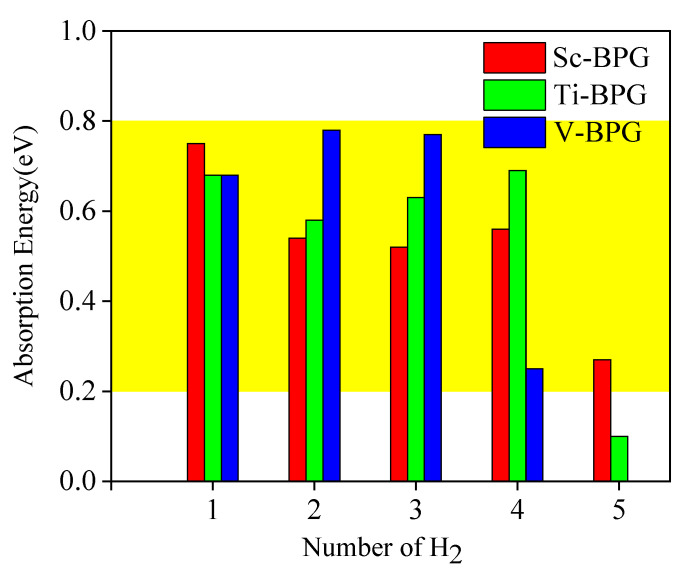
Adsorption energy of H_2_ molecules on transition metal-modified B-doped graphene (TM-BGr) system (yellow region represents the optimal energy adsorption range).

As shown in Figure 10, the adsorption energy parameters of H_2_ molecules in the Sc-BGr, Ti-BGr, and V-BGr systems are presented. The yellow region in the figure represents the optimal energy adsorption range (−0.2 to −0.8 eV) for efficient hydrogen storage [29,55]. Based on Figure 7A, Figure 8a and Figure 10, it can be observed that the Sc-BGr system is capable of reversibly and stably adsorbing up to 5 H_2_ molecules. The average adsorption energy is −0.528 eV/H_2_, which indicates a high hydrogen absorption rate. The hydrogen storage density of the Sc atom on BPG is the highest, at 1.54 wt.%, which is 25% higher than the hydrogen storage mass density of the other two metals (Ti, V) when they are modified on B-doped graphene. Therefore, the Sc-BGr system’s hydrogen storage performance is superior.

## 4. Conclusions

Based on first-principles DFT calculations, the geometric and electronic structures of Sc-, Ti-, and V-modified pure, B-doped, and N-doped graphene were investigated. The hydrogen storage performance of Sc-, Ti-, and V-modified B-doped graphene were studied. The results show that B doping can make the substrate lack electrons, which can effectively improve the interactions between Sc, Ti, and V and the graphene substrate. The modification sites of Sc, Ti, and V on B-doped graphene are all located at the hollow site of the carbon ring. Their adsorption energies are −3.21, −3.54, and −2.99 eV, respectively. Sc-, Ti-, and V-modified B-doped graphene can stably adsorb up to 5, 4, and 4 H_2_ molecules, respectively. The average adsorption energies are −0.528, −0.645, and −0.620 eV/H_2_. No dissociated H_2_ molecules are observed in all three systems. The electronic properties of the Sc-, Ti-, and V-modified B-doped graphene systems reveal that H_2_ molecules adsorb via both orbital interactions and polarization effects. The mass storage density of Sc-BSV is the highest at 1.54 wt.%, 25% greater than that of the other two metals (Ti, V). The findings of this work indicate that Sc-modified B-doped graphene stands out as an up-and-coming candidate for hydrogen storage applications.

## Figures and Tables

**Table 1:** Binding energies and related structural parameters of B/N-doped graphene modified by Sc, Ti, and V. The binding energy of the transition metal to substrate material ($E_{b}$); the distance between the transition metal and the atoms at Gr, BGr, and NGr are (TM-C), (TM-B), and (TM-N), respectively; the vertical distance between the transition metal and Gr, BGr, and NGr (H); and the Hirshfeld charge ($Q_{\mathrm{TM}}$) of transition metals.

Substrate	Adsorption Site	$E_{b}\;\left(\mathrm{eV}\right)$	TM-C (Å)	TM-B (Å)	TM-N (Å)	H (Å)	$Q_{\mathrm{TM}}\;\left(e\right)$
Sc-Gr	H	−1.34	2.374	-	-	1.893	+0.43
Sc-BGr		−3.21	-	2.412	-	1.892	+0.52
Sc-NGr		−1.45	-	-	2.270	1.822	+0.44
Ti-Gr		−1.80	2.333	-	-	1.842	+0.32
Ti-BGr		−3.54	-	2.349	-	1.806	+0.48
Ti-NGr		−1.86	-	-	2.208	1.769	+0.30
V-Gr		−1.30	2.315	-	-	1.820	+0.25
V-BGr		−2.99	-	2.314	-	1.757	+0.43
V-NGr		−1.06	-	-	2.106	1.684	+0.24

**Table 2:** Related parameters of H_2_ molecules on B-doped graphene modified by Sc, Ti, and V. The adsorption energy of H_2_ molecule ($E_{\mathrm{ads}}$); the average adsorption energy (${\overset{-}{E}}_{\mathrm{ads}}$); the distance between TM atom and H_2_ molecule ($d_{\mathrm{TM}-H_{2}}$); the closest distance between TM atom and C atom on B-Gr ($d_{\mathrm{TM}-C}$); and the bond length ($d_{H-H}$) of H_2_ molecule.

Substrate	$\mathrm{Number}\;\mathrm{of}\;H_{2}$	$E_{\mathrm{ads}}\;\left(\mathrm{eV}\right)$	${\overset{-}{E}}_{\mathrm{ads}}\;(\mathrm{eV}/H_{2})$	$d_{\mathrm{TM}-H_{2}}\;\left(Å\right)$	$d_{\mathrm{TM}-C}\;\left(Å\right)$	$d_{H-H}\;\left(Å\right)$
Sc-BGr	$1H_{2}$	−0.75	−0.750	2.008	2.353	0.837
	$2H_{2}$	−0.54	−0.645	2.062	2.396	0.808
	$3H_{2}$	−0.52	−0.603	1.927	2.450	0.860
	$4H_{2}$	−0.56	−0.593	2.012	2.474	0.814
	$5H_{2}$	−0.27	−0.528	2.420	2.477	0.765
Ti-BGr	$1H_{2}$	−0.68	−0.680	1.946	2.309	0.809
	$2H_{2}$	−0.58	−0.630	1.975	2.304	0.799
	$3H_{2}$	−0.63	−0.630	1.949	2.344	0.799
	$4H_{2}$	−0.69	−0.645	1.836	2.391	0.840
	$5H_{2}$	−0.0998	−0.536	4.701	2.394	0.752
V-BGr	$1H_{2}$	−0.68	−0.680	1.886	2.237	0.823
	$2H_{2}$	−0.78	−0.730	1.873	2.276	0.811
	$3H_{2}$	−0.77	−0.743	1.740	2.262	0.864
	$4H_{2}$	−0.25	−0.620	1.672	2.284	0.891
	$5H_{2}$	0.14	-	3.968	2.307	0.755

**Table 3:** Mulliken layout analysis before and after an H_2_ molecule adsorption by B-doped graphene modified with Sc, Ti, and V.

	Before Adsorption (e)			Charge (e)	After Adsorption (e)					Charge (e)
	Sc	Ti	V		Sc	Ti	V	H		
Sc-BGr	1.39	-	-	−1.39	1.86	-	-	−0.19	−0.16	−1.51
Ti-BGr	-	1.34	-	−1.34	-	1.63	-	−0.16	−0.10	−1.37
V-BGr	-	-	1.33	−1.33	-	-	1.57	−0.18	−0.16	−1.23

## Data Availability

The data presented in this study are available on request from the corresponding author. The data are not publicly available due to the extremely large size.
